# Impact of Different Drying Methods on the Phenolic Composition, In Vitro Antioxidant Activity, and Quality Attributes of Dragon Fruit Slices and Pulp

**DOI:** 10.3390/foods12071387

**Published:** 2023-03-24

**Authors:** Harsh Dadhaneeya, Radha Krishnan Kesavan, Baskaran Stephen Inbaraj, Minaxi Sharma, Srinivasulu Kamma, Prakash Kumar Nayak, Kandi Sridhar

**Affiliations:** 1 Department of Food Engineering and Technology, Central Institute of Technology, Kokrajhar 783370, India; 2 Department of Food Science, Fu Jen Catholic University, New Taipei City 242062, Taiwan; 3 Department of Applied Biology, University of Science and Technology Meghalaya, Baridua 793101, India; 4 Department of Food Technology, Koneru Lakshmaiah Education Foundation Deemed to be University, Vaddeswaram 522502, India

**Keywords:** novel drying technology, refractance window drying (RWD) method, total phenolic content (TPC), antioxidant activity (AA), total flavonoids content (TFC), physicochemical properties, quality attributes

## Abstract

The aim of this study was to compare the refractance window drying method (RWD) with the hot air oven drying (HD), vacuum drying (VD), and freeze-drying (FD) methods in order to analyze the outcomes of the qualitative properties of dragon fruit slices and pulp. Moreover, the impact of temperature on the phenolic content, antioxidant activity, color, and texture of the dragon fruit slices and pulp were studied. The results showed that the RWD samples exhibited a high nutritional quality in contrast to the other drying methods. The RWD method had a short drying time of 960 min to reach the final moisture content of 6.50% (dry basis), while the FD, VD, and HD methods had significantly higher drying times of 1320, 1200, and 1080 min, respectively, to reach the final moisture content. Higher values of TPC (182 mg GAE/100 g) and crude fiber (0.98%) were obtained in the RWD dragon fruit samples, indicating the potential of RWD to preserve the quality of dried samples. In conclusion, this study revealed that RWD provides an appropriate drying temperature as an alternative to freeze-drying. RWD may improve dragon fruit drying, adding value to the food industry.

## 1. Introduction

Dragon fruit, also known as pitahaya or pitaya, has a texture similar to kiwifruit due to its crunchy and edible black seeds found in the flesh. The dragon fruit seed oil contains primary fatty acids such as stearic acid, linoleic acid, and palmitic acid. Dragon fruit can be classified into four types based on flesh and skin color, including white dragon fruit (*Hylocereus undatus*), yellow dragon fruit (*Hylocereus selenicereus*), pink or violet dragon fruit (*Hylocereus costaricensis*), and red dragon fruit (*Hylocereus polyrhizus*) [1]. The red and purple flesh color is due to betacyanin pigments. Dragon fruit is a good source of fiber and magnesium, and it has a sweet, delicate taste. However, the high amount of water in it makes dragon fruit extremely perishable. That is the reason why drying this fruit is a challenging task.

Dragon fruit is a cactus fruit used in various foods, such as sherbet, syrup, and ice cream [2]. White dragon fruit is commonly produced in Southeast Asian countries such as Thailand, the Philippines, Vietnam, Indonesia, and southern China. Its high vitamin C content contributes to its excellent antioxidant effects, and its water-soluble fiber makes it refreshing and delicious. The skin or peel of dragon fruit is soft and pink in color, and it has the potential to be used as an antibacterial agent, a natural colorant, and an antioxidant [3]. Dragon fruit flesh is white and contains black edible seeds. The fruit has a light, sweet taste, and the amounts of antioxidants in white dragon fruit are less than those found in red dragon fruit. White dragon fruit is known to help prevent colon cancer and diabetes; stabilize the digestive process; and neutralize toxic compounds, especially heavy metals, and it may lower high blood pressure and cholesterol levels and even provide benefits against cough and asthma. Additionally, it is a rich source of potassium, protein, fiber, sodium, and calcium, making it a healthy fruit choice compared to other fruits [4].

In today’s world, consumers are increasingly seeking food products with higher nutritional value and a longer shelf life. Drying is an essential unit operation in the food industry and is the oldest method for extending the shelf life of food products by inhibiting microbial growth and enzymes. Dried products require less packaging, have better microbiological stability, last longer in storage, and weigh less when transported [5]. Conventional drying methods, including vacuum drying, hot air convective drying, microwave drying, and freeze-drying are widely used to enhance the product shelf life. However, in order to finish the drying process more effectively, integrated drying technologies are becoming more popular [6]. When selecting a drying method, the amount of energy used and the quality of the dried products are essential factors to consider. An efficient drying method that produces high-quality products is cost-effective since it reduces drying time and minimizes damage to the product. Drying is a very energy-intensive unit operation, accounting for about 20% of the total energy consumed in the food sector. Over 85% of commercial food dryers are convective, which use hot air as the heat transfer medium, resulting in significant product quality changes in the output dried product. Although food quality preservation is essential, energy efficiency is also a critical aspect of the drying process. Therefore, both factors are of utmost importance in designing, researching, and developing novel drying technologies [7].

Refractance window drying (RWD), one of the novel fourth-generation drying technologies, is used to dry heat-sensitive products. The RWD technology uses hot water at a temperature of 95 to 97 °C. The RW dryer transfers the heat energy of the hot water to the wet materials through a membrane. Generally, the actual temperature of the sample is 10 °C lower than the temperature of the hot water due to heat transfer losses between the water reservoirs and the external atmosphere [8].

Recently, refractive window drying has received considerable scientific attention due to its potential to produce high-quality dried products. However, understanding the drying mechanism and comparing it with conventional drying methods are important to expand its potential applications. This method is fast and used at moderate temperatures. Recent studies have used RW drying to obtain bone broth powder [9], goldenberry pulp [10], purple yam [11], pomegranate juice [8], sapota bars [12], aloe vera gel [13], cornelian cherry [14], yogurt [15], and passion fruit puree [16]. The RW method has become popular in the food sector due to the superior quality of the dried food, shorter drying time, absence of risk of cross-contamination, and low cost of the equipment compared to other dryers [17].

According to Nindo and Tang [18], the equipment and operational costs of the RW drier are one-third to half of that of the freeze dryer when drying the same product. The refractance window (RW) technology has successfully dried a wide variety of goods, including dairy ingredients, fruits, nutraceuticals, juices, and seafood [19]. The wet product is placed on the surface of the membrane, and the water in the product creates a “window” that allows thermal energy to travel through the material. This “window” permits heat radiation to pass through the material at the water–membrane–food interface [20]. The refractive index mismatch between the water and the food is significantly reduced at the point of contact, resulting in less reflection and increased thermal energy transfer into the food [21]. The RWD system offers better retention of phytochemical parameters compared to other drying technologies [22]. It is a new, energy-efficient, and eco-friendly drying technology that can benefit the food industry when selecting new drying technologies [23]. Small-scale industries can also easily implement this technology due to lower capital and maintenance costs. The research has shown that RWD output products have high nutritional and quality parameters, have high yields, and are non-hazardous. Many researchers have conducted RWD on various samples and reported that RW-dried products have a lower color change, higher rehydration ratio, higher total phenolic and total flavonoid contents, higher retention of total antioxidants, lower penetration force, and lower drying time compared to other convection drying samples. Along with energy consumption, product quality is also an essential factor in drying, which encompasses nutritional, functional, and sensory factors. The long drying time and high temperature affect product quality in conventional drying methods. Traditional drying methods involve cross-contamination due to the heat transfer media that enable direct interaction with the commodities. The RWD process is a type of film drying involving indirect interaction because the heating medium is not in direct contact with the product. Thus, the risk of contamination can be reduced [20]. The product’s appearance is also a crucial parameter in selecting suitable drying methods. Conventional drying methods have a lower drying rate, which is one of the main reasons why food products take a long time to dry. The high initial cost of industrial-scale dryers is also a significant issue for the food sector. Revolutionary RWD technology can address the difficulties encountered during drying. The present study was conducted to determine the effects of different drying methods on dragon fruit slices and pulp samples.

Therefore, the objective of this study was to evaluate the impact of different drying methods (RWD, HD, VD, and FD) on the drying curves, physicochemical parameters, and quality attributes (moisture content, color, texture, pH, TSS, ash content, crude fiber content, TPC, TFC, and antioxidant activities) of dragon fruit slices and pulp.

## 2. Materials and Methods

### 2.1. Sample Preparation

Fresh white dragon fruit (*Hylocereus undatus*) was purchased from a local fruit supplier in the Kokrajhar market, India. The selected fruits were between the maturation and ripening stage and weighed over 250 g. The peel needed to be a bright pink color, smooth, evenly colored, and free from wrinkles, while the flesh needed to appear juicy and free from any brown coloration, similar to watermelon. Based on these criteria, the dragon fruits were chosen and then washed with distilled water to remove dust and impurities. Next, the fruits were sliced 5 to 7 mm thick with a hygienic knife made of stainless steel. For pulp production, the dragon fruit slices were ground in a mixer and sieved using a 40-mesh size, with the resulting pulp spread out in a thin layer 5 to 8 mm thick. The samples were then ready for drying. After drying, the texture of the dried product was analyzed using a texture analyzer (TA-XT plus model, Micro-Systems, UK). Then, the dried product was ground into powder using a mixer grinder (410607, Bajaj Electricals Limited, India). The resulting powder was sieved and packed into LDPE pouches for further analysis. Fresh dragon fruits were taken as a control sample.

### 2.2. Operating Conditions of Drying

**Figure 1:**
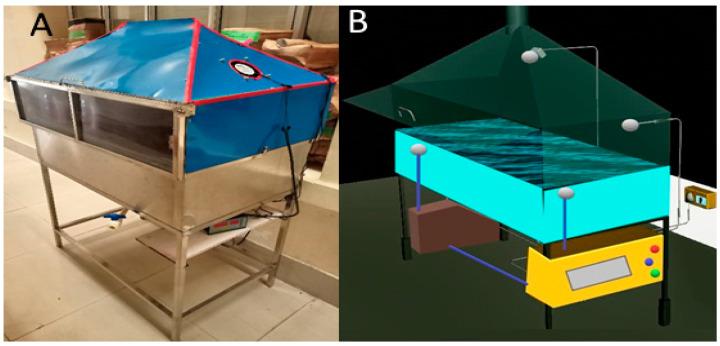
Refractance window drying (RWD) setup (**A**) and design (**B**).

This study employed four drying methods (RWD, HD, FD, VD) for drying dragon fruit slices and pulp samples. On a lab scale, a batch-type RW dryer was used for the drying experiments (Tacoma, Washington, USA). The RW dryer’s columns, mainframe, and water reservoir were all built from stainless steel because of the relatively low thermal conductivity of the stainless-steel alloy; this helped to lower the heat loss. The water reservoir was 99 × 61 × 27 cm^3^, and a mylar layer (PET—polyethylene terephthalate) membrane with a thickness of 0.09 mm was used as the membrane material. The RW dryer’s dimensions were 103 × 68 × 160 cm^3^, and the drying temperature for dragon fruit samples was maintained at 70 °C (water temperature) for drying. As the mylar layer can absorb or transmit the heat energy of water and reflect some of it, the temperature above the mylar layer reached 60 °C, indicating the sample was dried at 60 °C. The design of the RW dryer and the drying process are depicted in Figure 1. A lab-scale hot air oven (BTI-HAO-18 model, BioGene, Delhi, India) equipped with a PID control unit was used to dry the dragon fruit samples. The temperature was maintained at 60 °C and an airflow velocity of 2.0 m/s. The freeze dryer (Brog Scientific, India) was used for FD samples at a temperature of −18 °C and a pressure below 610 Pa. The moisture loss in the sample was checked every 2 h with an accuracy of ±0.01 g.

The laboratory vacuum oven (MSW-218, Macro Scientific Work Pvt. Ltd., Delhi, India) was used to prepare VD samples. A vacuum drying oven (MSW–218 lab-scale vacuum oven; Macro Scientific Work Pvt. Ltd., Delhi, India) with a cavity dimension of 345 mm by 370 mm by 415 mm was used for vacuum drying. The dragon samples were placed in a single layer on a glass culture dish and dried at 60 °C in an atmosphere of approximately 90 kPa when the moisture content of the samples dropped to 13% (wet basis).

### 2.3. Procedure of Preparing a Methanolic Extract Solution

Methanolic extract solutions were made as per the method given by Maduwanthi and Marapana [24]. Dried powder (1 g) was mixed with 35 mL methanol and then mixed for 3 min. After that, the sample was centrifuged for 10 min at 6026 × *g* (centrifuge rotor radius = 11 cm) and −18 °C (C-24BL, Remi Elektotechnik Ltd., New Delhi, India). After the centrifugation, the solution was filtered through filter paper. Lastly, the extracts were evaporated in a rotary evaporator at 40 °C. It was then ready for analysis and was stored in the refrigerator for further use.

### 2.4. Determination of Quality Parameters

#### 2.4.1. Moisture Content

The amount of moisture present in dried powder was established by drying the powder in a hot air oven at a temperature of 105 °C for 3 hr. The moisture content of the sample was evaluated in accordance with a predetermined protocol, AOAC (2000) [25].

#### 2.4.2. Texture Evaluation

The texture of the dried dragon fruit was determined using a texture analyzer (TA-XT plus model, Micro-Systems, UK). A 2.0 mm diameter cylindrical stainless-steel probe (P/2 probe) was used to compress the samples which were placed horizontally on the center point of the base plate. Here, the sample was compressed twice with a time interval of 5 s between two cycles. We set a value in the software, the pre-test speed was 1 mm/sec, the post-test speed was 5 mm/sec, the test speed was 5 mm/sec, the trigger force was set to 5 g, and the last penetration depth was set to 50% of sample thickness. Predefined values were obtained from Kek et al. [26]. The texture analysis software TEE 32 Stable Micro System (United Kingdom) was used to measure the sample’s hardness, springiness, cohesiveness, gumminess, and chewiness through the TPA (Texture Profile Analysis) curve.

#### 2.4.3. Color Analysis

The color of the samples was analyzed with the D-25LT model Hunter colorimeter, which Hunter Lab, USA manufactured. Following the method outlined by Nayak et al. [27], the color coordinates L^*^, a*, and b^*^ were instantly captured as the mean of 3 observations at different locations of the samples. A white calibration plate with the values L = 90.55, a = −0.71, and b = 0.39 was used to calibrate the apparatus before any measurements were carried out.

#### 2.4.4. pH, Total Ash Content, and Total Soluble Solids (TSS)

To measure the pH and TSS, first, 10,000 µL of distilled water was added to 1000 mg of sample powder and shaken well in a spinix vortex shaker for complete dilution. The ION 2700 model pH meter was used for pH measurements. AOAC (2000) [25] standard procedure was followed for pH measurements. TSS was determined using a refractometer according to AOAC (2000) [25] standard procedure. The % ash of dried powder samples was determined as per the published standard procedures of AOAC (2000) [25] by using a muffle furnace (NSW-101, Narang Scientific Works Pvt. Ltd., New Delhi, India).

#### 2.4.5. Crude Fiber Content

The amount of crude fiber was ascertained according to the published standard procedures of AOAC (2000) [25] using the FES04E model crude fiber analysis apparatus (Pelican Equipment, India). Briefly, a crucible containing 1 g of sample was placed into the crude fiber testing instrument after the sample had been weighed accurately. To analyze the sample, the sample was first heated in a solution of sulphuric acid and then in a solution of sodium hydroxide; both had a concentration of 1.25%. The specimen was then subjected to a hot air oven at 100 °C for 2 h. Then, the dried sample was put in a muffle furnace at 55 °C for 4 h and weighed. The crude fiber content was expressed as %.

#### 2.4.6. TPC

The Folin–Ciocalteu reagent method, described by Kupina et al. [28], was implemented in order to conduct the analysis that determined the total amount of phenolics present in the sample. In a separate container, 500 µL of methanolic extract was mixed with 500 µL of Folin and Ciocalteu’s phenol reagent and 7500 µL of distilled water. After waiting 3 min, 1500 µL of a sodium carbonate solution of 20% *w*/*v* was added to the mixture, and the total volume was brought up to 10,000 µL. After allowing the reaction to proceed in the dark for the allotted time (60 min), an absorbance reading was taken at 765 nm using the model 2375 UV-V spectrometer (Electronics India, Haryana, India). The amounts of phenols in the test samples were determined using the calibration curves of gallic acid. The results represented the quantity of mg gallic acid equivalent per 100 g of sample.

#### 2.4.7. TFC

The aluminum chloride procedure that was specified in Khatiwora et al. [29] was adopted in order to ascertain the total flavonoid content of the dragon fruit sample extracts. Methanolic extract (6000 µL) was mixed with 200 µL of 10% AlCl_3_ and Na-K tartrate solution (200 µL). Then, 5600 µL of distilled water was inserted and vortexed for 30 min. The absorbance was read at 415 nm using a UV-V spectrometer. The quercetin concentrations in the test samples were determined by using the quercetin calibration curve and are reported as the amount of quercetin present in milligrams per gram of the sample.

#### 2.4.8. DPPH Radical Scavenging Activity

The approach that Saikia et al. [30] describe was utilized in order to ascertain the level of DPPH radical scavenging activity. Firstly, the DPPH solution was processed by dissolving 0.004 µg of DPPH in 100 mL of methanol. Methanolic extract (400 µL) was mixed with 5600 µL of DPPH solution. The mixture was shaken, stored in a dark place, and allowed to stand at room temperature for 30 min. A UV-V spectrometer was used in order to determine the absorbance of the final solution at a wavelength of 515 nanometers.

#### 2.4.9. FTIR Spectra

Dried dragon fruit powder obtained using various drying treatments was evaluated by Fourier-Transform Infrared Spectrometry according to Zhao et al. [31]. The dragon fruit powder samples were prepared with the potassium bromide (KBr) pellet method. Reference spectra were taken under identical conditions, and the KBr medium with no powders was used as a blank. The spectra were corrected for baseline in the region of 400–4000 cm^−1^ using Origin 2022b software (Origin Lab, Northampton, MA, USA).

### 2.5. Statistical Analysis

The outcomes are presented in the form of mean values ± standard deviation values of triplicates. A statistical analysis was performed on the data using a 1-way ANOVA in conjunction with Tukey’s tests for genuinely significant differences at a significance level of *p* < 0.05 using the Origin 2022b software.

## 3. Results

### 3.1. Moisture Content (MC)

**Figure 2:**
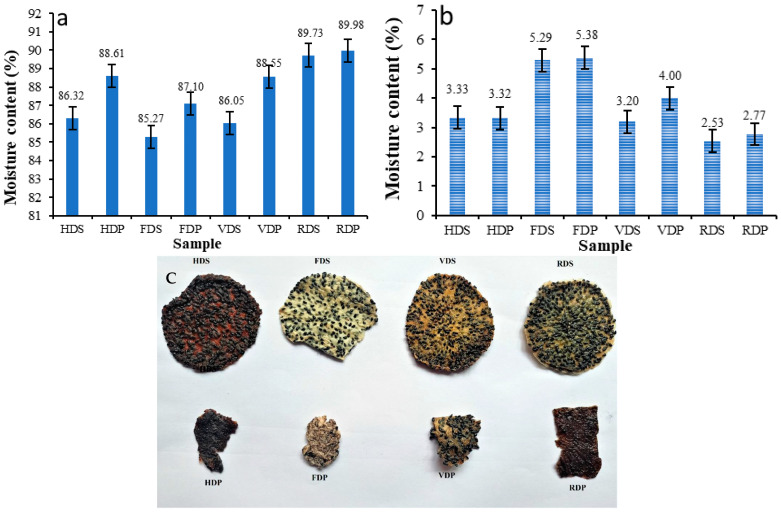
Moisture content of (**a**) dragon fruit sample and (**b**) dried sample powder, and (**c**) the dried products. HDS, hot air oven-dried dragon fruit slice; HDP, hot air oven-dried dragon fruit pulp; FDS, freeze-dried dragon fruit slice; FDP, freeze-dried dragon fruit pulp; VDS, vacuum-dried dragon fruit slice; VDP, vacuum-dried dragon fruit pulp; RDS, RW-dried dragon fruit slice; RDP, RW-dried dragon fruit pulp.

When fresh and ripe, dragon fruits are highly perishable due to their high moisture content. Figure 2a shows the amount of moisture present in the dragon fruit slice and pulp samples under different drying conditions. The MC of the dragon fruit slice samples dried by HD, FD, VD, and RWD were found to be 86.32%, 85.27%, 86.05%, and 89.73%, respectively. The moisture content of the pulp samples dried by HD, FD, VD, and RWD was found to be 88.61%, 87.10%, 88.55%, and 89.98%, respectively. From the figure, it can be observed that the FD samples have a lower moisture content compared to the other dried samples. This may be due to that fact that FD is more efficient in decreasing the moisture level of the food material which has a high water content [32]. The pulp sample had a higher MC value compared to the slice sample due to the grinding and sieving unit operations carried out in the sample preparation, which resulted in microscopic and uniform particles. Consequently, heat waves penetrated the pulp more easily than the slices. To obtain accurate and appropriate results, the slice and pulp thicknesses were kept equal at 5 to 7 mm. The MC value of dragon fruit flesh was found to be 83% in the study conducted by Jaafar et al. [4], which is nearly equal to the values obtained in the present study.

The dried powders were obtained by grinding the products that were dried using the aforementioned drying methods, and their moisture content is presented in Figure 2b. The MC of sliced–dried dragon fruit powder produced by HD, VD, FD, and RWD was found to be 3.33%, 5.29%, 3.20%, and 2.53%, respectively. Similarly, the moisture content of pulp dried powder samples produced by HD, FD, VD, and RWD was found to be 3.32%, 5.38%, 4.00%, and 2.77%, respectively. The dried products are shown in Figure 2c.

### 3.2. Drying Characteristics of Dragon Fruit Sample

**Figure 3:**
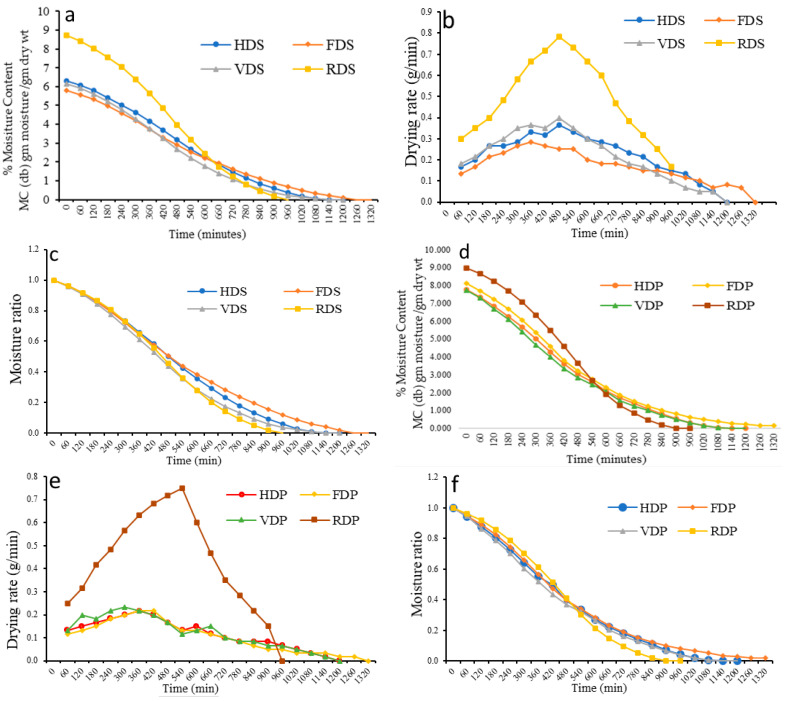
Graphical representation of slice samples: (**a**) % MC vs. drying time, (**b**) drying rate vs. drying time, (**c**) moisture ratio vs. drying time, and of pulp samples: (**d**) % MC vs. drying time, (**e**) drying rate vs. drying time, and (**f**) moisture ratio vs. drying time. MC, moisture content.

The initial MC of the dragon fruit slice samples ranged from 5.789 to 8.737 MC (db) g moisture/g dry wt, while for the pulp samples it ranged from 7.374 to 8.980 MC (db) g moisture/g dry wt. Figure 3 depicts the moisture content vs. drying time, drying rate vs. drying time, and moisture ratio vs. drying time of the dragon fruit samples for the slice samples Figure 3a–c and pulp samples Figure 3d–f. The drying rate of RWD was found to be higher than that of other drying methods, leading to some variation in drying time values. Similar results were obtained by Krishnan et al. [33] while drying elephant apple (*Dillenia indica*) slices under various drying conditions. The drying rate of the pulp samples was higher in all the drying methods compared to the slice samples. The drying rate value obtained for the RWD sample was 0.750 g/min at 540 min for the pulp and 0.783 g/min at 480 min for the slice samples. When comparing the drying rates of the pulp and slice samples at the same time, the drying rate of the sliced samples was higher. The drying curves of the freeze-dried samples were progressively lower than those of the other methods. In contrast, the drying curves of the RWD samples were higher than those of the other methods. Harsh et al. [34] reported similar findings for Malbhog bananas.

### 3.3. pH, Ash Content, TSS, Crude Fiber, and Drying Time

The optimum pH of fresh dragon fruit, as reported by Jalgaonkar et al. [3], falls in the range of 3 to 7. In this study, the control sample (fresh dragon fruit) had a pH of 7.96, and the pH meter showed the pH of all dried dragon fruit samples to be between 6.23 and 7.86, with a significant change in pH values observed with increasing temperature, as presented in Table 1. The pH value was highest in the FD samples and lowest in the HD samples, and the pH of the pulp samples was found to be higher than that of the slice samples. The RDS sample also had a higher pH value than the FDS sample.

Ash content fluctuated slightly among the drying methods, ranging from 0.84% (FDP) to 1.54% (HDS). The highest total ash content of 1.54% was found in the HD samples, followed by the RWD samples, while the lowest value of 0.84% was found in the FD samples. Notably, the freeze-dried powder contained a higher amount of free water, and that evaporated during the ash content determination process in the muffle furnace due to the high temperature. The slice sample had a higher ash content than the pulp sample, with the control sample having the lowest ash content. Here, the total ash content was taken on a dry basis. Similar observations were made by Jaafar et al. [4] regarding the fluctuation in total ash content of dragon fruit stem.

According to Abirami et al. [35], the TSS of dragon fruit ranges from 9.1 to 18.3 °Brix, while in this study the control sample had 13 Brix, and for the dried samples the TSS ranged from 9 to 12 Brix. On the other hand, no significant difference was found in the TSS of the dried dragon fruit samples. TSS values were the same for the HD and RD samples. The RDP and HDP samples (12 Brix) had the highest TSS, and the lowest TSS was in the FD samples (9 Brix), as shown in Table 1. The TSS value of the FD samples was lower compared to the other dried samples. It is possible that the oxidative degradation of complex materials such as organic acid, starch, and sugars occurs due to exposure during heat treatment and direct interaction with a heat transfer medium [36].

After the control sample, the RWD sample had higher crude fiber content values, which were 0.96 and 0.98 for the slice and pulp samples, respectively. The FD sample had the lowest value because the fiber content may have been decreased due to the application of a lower temperature during processing. The increase in the crude fiber content with the RWD method can be explained by the higher temperature during drying that may break down the cellular matrix of the dragon fruit slices. The same observation has been noted in the studies of Adeboye et al. [37] and Ho et al. [38]. Further, it can be stated that the crude fiber content in the dragon fruit can be elevated by the use of higher drying temperatures which may disintegrate the pectin present in the slices. The pulp sample had a higher value of crude fiber content than the slice sample. Jaafar et al. [4] also determined a similar crude fiber content in the dragon fruit sample.

RWD was found to take much less time to dry the dragon fruit, around 960 min, compared to the other drying methods, which took 1200 to 1320 min. This reduction in drying time highlights RWD’s unique and speedy heat transfer mechanism. Table 1 shows the drying times of the dragon fruit samples.

### 3.4. Texture Analysis

When comparing the drying methods, the textural properties were better for the RW-dried samples because of the uniform and controlled drying process in the RW dryer, which resulted in well-dried samples being obtained. However, apart from springiness and cohesiveness, all other texture analysis property values were higher in the slice samples than in the pulp samples, likely due to the grinding operation during sample preparation that produced uniformly distributed particles in the pulp samples. Another possible reason could be the crushing of black seeds in the fruit pomace during the grinding process followed by the sieving process, which removed the big particles of the seeds and the remaining whole seeds that had not been crushed. The RWD samples had hardness values of 1089.33 for slices and 101.95 for pulp, which were substantially higher than the samples dried using other methods. This higher hardness value for the RWD sample indicates that the sample was adequately dried in the RWD system. For the slice sample, the values of springiness were 0.83, 0.64, 0.71, and 0.98 for the HDS, FDS, VDS, and RDS samples, respectively. For the pulp sample, the values of springiness were 1.08, 1.02, 0.73, and 1.11 for the HDP, FDP, VDP, and RDP samples, respectively. With regard to both the pulp and slice samples, the RWD sample obtained a higher springiness value (0.982 for slice and 1.118 for RDP) in comparison to the other samples. There was a disparity in the chewiness of the sliced HDS, FDS, VDS, and RDS samples which had values of 234.98, 119.04, 468.41, and 741.24, respectively. Similarly, the HDP, FDP, VDP, and RDP samples all had chewiness values of 112.20, 2.04, 42.208, and 102.04, respectively. For the slice sample, the adhesiveness values for the HDS, FDS, VDS, and RDS samples were 0.18, −0.273, −2.544, and −5.20 g.sec, respectively. For the pulp sample, the adhesiveness values for HDP, FDP, VDP, and RDP were −1.791, −5.19, −0.667, and −1.213 g.sec, respectively. The resilience values of the slice samples were 0.25, 0.21, 0.19, and 0.288 for the HDS, FDS, VDS, and RDS samples, respectively. Moreover, the resilience values of the HDP, FDP, VDP, and RDP samples were 0.36, 0.26, 0.213, and 0.328, respectively.

### 3.5. Color Analysis

Drying is known to cause inappropriate changes in the primary color of the dried product, which can have a significant impact on consumer acceptability. Table 1 shows the color changes in the dragon fruit samples resulting from the different drying methods. The L, a, and b values of the fresh dragon fruits were measured as 66.23 ± 1.45, 1.36 ± 0.25, and 5.97 ± 0.95, respectively. The RWD samples had L, a, and b values of 42.48, 5.18, and 29.58 for the slice sample and 24.70, 15.58, and 13.54 for the pulp sample, respectively. The Δ E value of the RWD slice and pulp samples were similar at 48.12 and 46.64, respectively. The FD sample exhibited a brighter color, indicating the highest L value (71.05 for FDS samples and 46.60 for FDP samples) compared to the HD, VD, and RW-dried samples. In contrast, the HD and VD samples had the lowest L values, indicating a darker product color. The high temperatures used in HD and VD may have caused instability and degradation of the color pigments, leading to the acceleration of Maillard and enzymatic reactions or caramelization processes and resulting in the formation of brown pigments. The pulp sample had a higher L value than the slice sample. For the slice sample, a^*^ values were recorded as 7.91, −2.37, 9.41, and 5.18 for HDS, FDS, VDS, and RDS, respectively. For the pulp sample, a^*^ values were observed as 1.74, 4.69, 7.1, and 15.58 for HDP, FDP, VDP, and RDP, respectively. The b* values recorded for the slice sample were 6.08, 20.32, 38.31, and 29.58 for the HDS, FDS, VDS, and RDS samples, respectively. Similarly, the b* values recorded for the pulp sample were 0.627, 14.16, 16.46, and 13.54 for the HDP, FDP, VDP, and RDP samples, respectively. Baeghbali et al. [8] found similar changes in L, a, and b values when drying pomegranate juice using RWD, spray drying, and FD. However, in their study, most of the a and b values were opposite to the L values.

### 3.6. TPC, TFC, and AA

**Figure 4:**
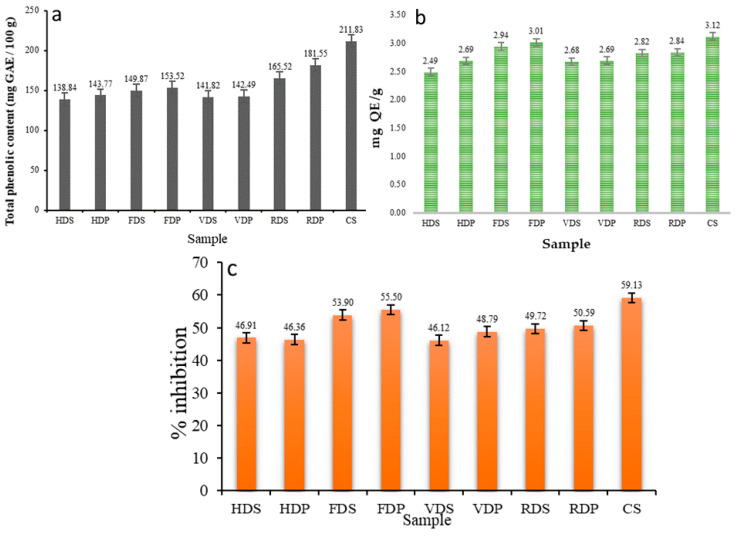
Total phenolic content (**a**), total flavonoid content (**b**), and antioxidant activity (**c**) of dragon fruit samples. For sample codes, refer to Figure 2.

The TPC in the control sample was 211.83 mg GAE /100 g. The RWD samples had the highest TPC (165.52 mg GAE/100 g for the RDS and 181.55 mg GAE/100 g for the RDP samples) values after the control sample (Figure 4a), which were extremely close to the FD samples (149.87 mg GAE/100 g for FDS and 153.52 mg GAE/100 g for FDP), whereas the lowest TPC was observed in the HD (138.84 mg GAE/100 g for the HDS and 143.77 mg GAE/100 g for the HDP samples) and VD (141.82 mg GAE/100 g for the VDS and 142.49 mg GAE/100 g for the VDP samples) samples. The reduction in TPC content in the dried dragon fruit products could be due to the degradation (oxidation) of phenolic compounds during thermal treatment, especially in the HD and VD samples because of the longer drying time [39]. The TPC value of the slice sample was slightly lower than that of the pulp sample.

The results of TFC expressed in terms of QE/g of a sample are presented in Figure 4b. The control sample had a TFC value of 3.12 mg QE/g. As for the TPC, the FD sample had the maximum TFC values (2.94 mg QE/gm for FDS and 3.01 mg QE/g for FDP) after the control sample, and the RWD sample had the second-highest value. The TFC values were 2.82 and 2.84 mg QE/g for the slice and pulp samples, respectively. The TFC value was higher in the FD samples than in other drying methods, mainly due to the lower temperatures of FD, which contribute to the preservation of flavonoid compounds. Feng et al. [40] reported similar changes in values in apple samples, and Chen et al. [41] identified eight flavonoid subgroups in Australian-grown white dragon fruits, including dihydro flavonols, anthocyanins, flavanones, dihydrochalcones, flavones, flavonols, and isoflavonoids.

The AA of the control sample was recorded as 59.13%, as displayed in Figure 4c. The values of AA were highest in the FD sample, with values of 53.90 and 55.50 for the slice and pulp samples, respectively. The main reason for this is that the lower drying temperatures in FD help to preserve the bioactive compounds. The RWD sample had the second-highest value (49.72% for RDS and 50.59% for RDP). The systematic rupture of cell walls during uniform and controlled drying temperatures in RWD, HD, and VD gradually activates the release of oxidative and hydrolytic enzymes, resulting in the degradation of bioactive compounds in the dry product [42]. The AA value of the control sample was 59.13%. The pulp sample had a higher antioxidant activity than the slice sample, possibly because the pulp homogenization led to cell rupture and facilitated the release of bioactive compounds. Greater heating in HD, VD, and RWD at a temperature of 60 °C assisted the breakdown of cell components, resulting in a significant decrease in antiradical activity. Dal-Bó and Freire [43] obtained similar results in avocados (*Persea Americana*).

### 3.7. FTIR Spectroscopy

**Figure 5:**
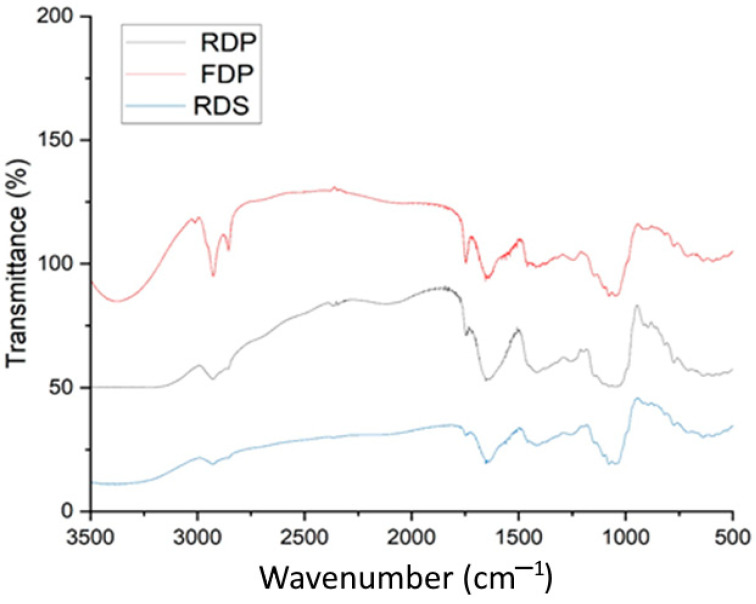
FTIR analysis of dragon fruit samples. For sample codes, please refer to Figure 2.

FTIR spectroscopy was used to assess the modifications that were produced by a variety of drying methods in the functional groups. Figure 5 illustrates a total of four primary absorbance peaks, which were located at 2927 cm^−1^ of the regions of 3000 to 2500 cm^−1^, referred to as the C-H group of alkanes; 1745 cm^−1^ of the zone of 2000 to 1500 cm^−1^ attributed to the C=O group of cyclopentanone, and in this same region the 1638 cm^−1^ peak was also observed which referred to the C=C group of alkenes; and 1045 cm^−1^ of the zone of 1500 to 1000 cm^−1^ referred to as the CO-O-CO group of anhydrides. Moreover, some weak points can be seen in the zone of 800 cm^−1^ to 650 cm^−1^, referred to as the C-H groups.

## 4. Conclusions

The impact of utilizing various drying methods (HD, FD, VD, and RWD) on the quality properties of dragon fruit slice and pulp samples was analyzed. The study revealed that the RWD method could best preserve the physical and chemical properties compared to the other drying methods. RW-dried samples had better overall color and texture, as well as greater TPC and crude fiber than samples dried using other methods. The result of the study showed that due to the higher drying rate of RWD, the dragon fruit sample took 960 min to dry, which is a significantly shorter period than for the other drying methods. In comparison to the RWD samples, the FD samples had the highest TFC values and % antioxidant activity but required a longer drying time. The RWD method has the potential for drying fruits and vegetables with acceptable product quality, lower production costs, less thermal damage, and shorter processing times. RWD may have a promising future in meeting the stringent performance and quality requirements for drying heat-sensitive commodities.

## Figures and Tables

**Table 1:** Physicochemical, drying time, and quality attributes of dragon fruit samples ^1^.

Parameters	HDS	HDP	FDS	FDP	VDS	VDP	RDS	RDP	Control
Drying time (min)	1200	1200	1320	1320	1200	1200	900	840	-
Ash (%)	1.54 ± 0.02 ^a^	1.48 ± 0.03 ^b^	0.89 ± 0.01 ^ef^	0.84 ± 0.02 ^ef^	1.06 ± 0.03 ^g^	0.95 ± 0.04 ^d^	1.49 ± 0.01 ^cd^	1.34 ± 0.01 ^de^	0.65 ± 0.02 ^h^
pH	6.23 ± 0.01 ^g^	6.57 ± 0.02 ^c^	6.78 ± 0.01 ^f^	7.86 ± 0.01 ^b^	6.78 ± 0.01 ^f^	7.05 ± 0.01 ^d^	6.89 ± 0.02 ^e^	7.42 ± 0.01 ^da^	7.96 ± 0.025 ^h^
TSS (°Brix)	10.5 ± 0.2 ^bcde^	12 ± 0.2 ^ab^	9 ± 0.15 ^efgh^	10 ± 0.2 ^cdef^	9 ± 0.2 ^cd^	9.5 ± 0.2 ^bc^	10.5 ± 0.2 ^ef^	12 ± 0.2 ^bc^	13 ± 0.2 ^f^
Crude fiber (%)	0.86 ± 0.02 ^d^	0.91 ±0.22 ^cd^	0.72 ± 0.02 ^bc^	0.78 ±0.02 ^b^	0.82 ± 0.16 ^cd^	0.86 ± 0.02 ^bcd^	0.96 ± 0.02 ^bc^	0.98 ± 0.38 ^a^	1.14 ± 0.15 ^g^
**Texture**									
Hardness (g)	450.43 ± 5.62 ^a^	139.131 ± 3.21 ^b^	244.041 ± 4.32 ^ac^	23.60 ± 2.62 ^cd^	723.596 ± 8.62 ^c^	84.612 ± 3.41 ^d^	1089.33 ± 7.63 ^c^	101.959 ± 3.41 ^d^	18.952 ± 2.75 ^e^
Fracturability (g)							538.23 ± 16.32 ^a^		
Adhesiveness (g.sec)	0.18 ± 0.026 ^a^	−1.791 ± 0.12 ^ba^	−0.273 ± 0.076 ^ac^	−5.191 ± 0.12 ^d^	−2.544 ± 0.099 ^e^	−0.667 ± 0.031 ^f^	−5.220 ± 0.16 ^g^	−1.213 ± 0.11 ^e^	−0.089 ± 0.009 ^d^
Springiness	0.839 ± 0.12 ^d^	1.088 ± 0.92 ^c^	0.643 ± 0.098 ^g^	1.029 ± 0.45 ^cd^	0.714 ± 0.11 ^dg^	0.735 ±0.16 ^f^	0.982 ± 0.26 ^b^	1.118 ± 0.63 ^ab^	0.04 ± 0.0084 ^e^
Cohesiveness	0.622 ± 0.11 ^cd^	0.741 ± 0.099 ^ef^	0.488 ± 0.076 ^g^	0.842 ± 0.11 ^d^	0.647 ± 0.16 ^cd^	0.678 ± 0.22 ^d^	0.680 ± 0.10 ^b^	0.895 ± 0.16 ^a^	0.256 ± 0.043 ^f^
Gumminess	279.98 ± 8.36 ^c^	103.111 ± 3.45 ^cd^	119.048 ± 4.56 ^d^	1.988 ± 0.12 ^cd^	468.41 ± 5.625 ^f^	57.403 ± 1.23 ^bc^	741.246 ± 7.65 ^c^	91.302 ± 2.36 ^a^	109.86 ± 7.32
Chewiness	234.98 ± 4.56 ^a^	112.209 ± 3.69 ^d^	76.531 ± 1.63 ^b^	2.04 ± 0.765 ^d^	334.58 ± 7.89 ^g^	42.208 ± 6.54 ^d^	728.010 ± 11.12 ^a^	102.044 ± 3.69 ^b^	32.16 ± 1.25 ^g^
Resilience	0.250 ± 0.07 ^bc^	0.360 ± 0.083 ^a^	0.210 ± 0.062 ^ab^	0.26 ± 0.073 ^a^	0.19 ± 0.074 ^c^	0.213 ± 0.084 ^e^	0.288 ± 0.087 ^f^	0.328 ± 0.076 ^d^	0.162 ± 0.088 ^h^
**Color value**									
L*	16.28 ± 1.25 ^da^	23.46 ± 1.23 ^ce^	71.05 ± 4.32 ^c^	46.60 ± 2.52 ^a^	49.36 ± 2.65 ^b^	33.46 ± 1.56 ^ef^	42.486± 2.98 ^d^	24.70 ± 1.15 ^b^	66.23 ± 1.45 ^ac^
a*	7.91 ± 0.89 ^c^	1.74 ± 0.64 ^dg^	−2.37 ± 0.88 ^bc^	4.69 ± 0.99 ^d^	9.41 ± 1.77 ^c^	7.1 ± 1.07 ^bc^	5.18 ± 0.88 ^a^	15.58 ± 1.01 ^c^	1.36 ± 0.25 ^g^
b*	6.08 ± 0.97 ^e^	0.627 ± 0.076 ^df^	20.32 ± 1.25 ^eg^	14.16 ± 1.06 ^b^	38.31 ± 2.69 ^a^	16.46 ± 1.06 ^d^	29.58 ± 1.99 ^c^	13.54 ± 1.01 ^c^	5.97 ± 0.95 ^ae^
∆ E	60.09 ± 3.12 ^b^	59.95 ± 3.04 ^cd^	29.69 ± 1.19 ^d^	24.17 ± 1.97 ^a^	59.50 ± 2.76 ^ab^	59.41 ± 3.87 ^cd^	48.12 ± 2.99 ^cd^	46.64 ± 4.46 ^ac^	-

^1^ Data presented as mean value ± standard deviation. Row with the equal superscript letters indicates not significantly different at *p* < 0.05. For sample codes, refer to Figure 2.

## Data Availability

Data are contained within the article.
